# Case Report: A 1-year progression of mediolateral gait instability during tandem walking in FXTAS

**DOI:** 10.3389/fspor.2025.1637831

**Published:** 2025-10-21

**Authors:** Jonathan Lee-Confer, Dian Baker, Randi Hagerman, Nell Maltman, Megan Kobel, Rodney Imamura

**Affiliations:** ^1^ Department of Kinesiology, California State University, Sacramento, CA, United States; ^2^ Department of Physical Therapy, University of Arizona, Tucson, AZ, United States; ^3^ Department of Physiology, University of Arizona, Tucson, AZ, United States; ^4^ Medical Investigations of Neurodevelopmental Disorders Institute, Sacramento, CA, United States; ^5^ Department of Nursing, California State University, Sacramento, CA, United States; ^6^ Department of Speech Language and Hearing Sciences, University of Arizona, Tucson, AZ, United States

**Keywords:** fragile x-associated tremor/ataxia syndrome (FXTAS), gait, step width, tandem walking, longitudinal, follow-up, variability, balance impairment

## Abstract

Fragile X-associated Tremor/Ataxia Syndrome (FXTAS) is a late-onset neurodegenerative disorder characterized by progressive motor dysfunction, including cerebellar ataxia and gait instability. Although tandem walking is a sensitive clinical marker of cerebellar dysfunction, its utility in tracking longitudinal motor decline in FXTAS remains unexplored and the trajectory of motor decline in FXTAS is not well characterized. Therefore, the purpose of this case report was to determine whether tandem walking performance deteriorates over a one-year period in an individual with FXTAS. A 68-year-old male with genetically confirmed FXTAS completed a 15-second tandem walking trial at baseline and again after one year. Kinematic data were collected using a Vicon motion capture system. Step width was calculated at each heel strike as the distance between the mediolateral position of the left and right heel markers. The mean step width considerably increased from baseline tandem walking of 45.21 ± 33.47 mm (SD) compared to the 1-year follow-up trial step width of 85.79 ± 15.80 mm (SD) indicating potential progressive mediolateral instability. This case report provides preliminary evidence that step width during tandem walking may be a sensitive marker of longitudinal motor decline in FXTAS and declines in gait stability can occur within one year. Larger studies with repeated measures and additional gait metrics are warranted to validate these findings.

## Introduction

Gait is often used as one of the metrics to determine the quality of life of an individual due to the importance of mobility on everyday functioning (1–3). Metrics such as gait speed, step width, step length, and cadence have been used to compare pathological to healthy controls to better understand characteristics of neurological conditions (4–7). Gait deviations have been extensively studied in populations such as stroke (8–10), ataxia (11–13), Parkinson's disease (14–16), and cerebral palsy (17–19). Perhaps more importantly than classifications of gait by neurological disorders, the progression of neurological conditions as they manifest as gait deviations have been studied and reported (20–23). A less studied neurological degenerative disorder known as Fragile X-associated Tremor/Ataxia Syndrome (FXTAS), has received less attention in the analysis of biomechanical gait studies. The prevalence of the fragile X premutation is 1 in 150–300 females and 1 in 400–850 males with majority of the males with the premutation developing FXTAS (24). FXTAS is characterized by cerebellar ataxia, intention tremor, cognitive decline, and neural changes seen on MRI (i.e., middle cerebellar peduncle sign representing white matter disease, and global brain atrophy) (24). Characterizing gait changes across the progression of neurological disorders such as FXTAS, Parkinson's disease, and cerebellar ataxias is essential, as gait impairments often reflect the underlying neurodegenerative process and may serve as early biomarkers of disease severity and progression. Understanding these temporal changes can inform clinical staging, guide intervention strategies, and improve fall risk management across disease stages.

The current understanding of walking gait in FXTAS is limited as this disease was relatively recently discovered (25). Compared to healthy controls, individuals with FXTAS exhibited significantly impaired gait across all domains, characterized by reduced stride velocity and cadence, increased gait variability, and longer double-limb support times (26). Furthermore, individuals with FXTAS exhibited a reduced stride velocity, increased stride variability and asymmetry (27). Prior work provides insights into gait deficits in individuals with FXTAS, however the progression of the disease's effect on gait remains elusive.

A common hallmark gait deviation in individuals with ataxia is an increased step width (5, 28, 29). Tandem walking in individuals with ataxia becomes an increasingly difficult task with increased step width, high variability in foot placement, and a significantly greater number of missteps compared to controls (28). As such, tandem walking is one of the most sensitive clinical measurements to detect cerebellar dysfunction (30). Furthermore, step width may be an indicator of cerebellar health during tandem walking as individuals with cerebellar disease did not differ in gait speed, step length, cadence, step height, foot angle, stance time, or swing them when compared to healthy controls (28).

Therefore, the purpose of this case report was to investigate whether tandem walking could detect progression in gait deficits after a one-year follow-up period in an individual with FXTAS. We hypothesized that the participant would exhibit a wider step width at the one-year follow-up compared to baseline during a 15-second tandem walking trial.

This investigation serves as a feasibility report to determine whether quantitative tandem gait assessment can detect longitudinal changes in motor performance in FXTAS, and to evaluate the practicality of using step width as markers of disease progression in a single-subject design. Establishing reliable markers of gait progression, such as changes in tandem walking performance, could lay the groundwork for defining distinct stages of motor decline in FXTAS and lead to larger study sizes. By complementing tandem gait assessment with standard gait metrics over time, it may be possible to develop a comprehensive, clinically relevant staging system that reflects the natural trajectory of gross motor impairment in this population.

## Methods

### Participant

One participant was recruited from the Medical Investigations of Neurodevelopmental Disorders (MIND) Institute and was medically confirmed through genetic testing to have the premutation of the fragile-X messenger ribonucleoprotein 1 gene (*FMR1*) and confirmed FXTAS. FXTAS was diagnosed genetically by the MIND Institute. At baseline, the participant was a 68 year old male, 177 cm in height, weighed 110 kg, and had a BMI of 35.1. The participant's FXTAS stage was unknown at the time of the biomechanical testing. The gait evaluation of this FXTAS case report was not part of a standard procedure at the Medical Investigation of Neurological Disorders Institute as only one individual's data was collected. We believe the individual was in the earlier stages of the disease due to independence of ambulation but this was not officially determined. Furthermore, the participant was able to drive independently to our laboratory. Gait analysis of the participant was conducted at the California State University, Sacramento in the Biomechanics Laboratory. The participant signed an informed consent that was approved by the Institutional Review Board at the California State University, Sacramento (31). Follow-up testing of gait for the FXTAS participant occurred one year later at the age of 69 years.

### Instrumentation

An 8-camera Vicon motion capture system (Vicon 612, Vicon Motion Systems, Lake Forest, CA, USA) collected kinematic data at 100 Hz at the California State University, Sacramento.

### Procedures

The participant had reflective markers placed bilaterally on the posterior heel on the calcaneus, in line with the Achilles tendon. The participant was instructed to perform a single 15-second tandem walking trial by taking steps and having each foot land in front of the other foot at their self-selected speed. The tandem walk was visually demonstrated by the researcher. The participant did not complete a familiarization trial, but did practice taking 2 steps for confirmation that he was performing this correctly. The 15-second data collection began when the participant took their first tandem step. The participant traversed a designated 6-meter walkway and completed a single trial, during which they took as many steps as possible within the allotted time. The trial ended after 15 s, regardless of how far the participant traversed.

### Data analysis

**Figure 1:**
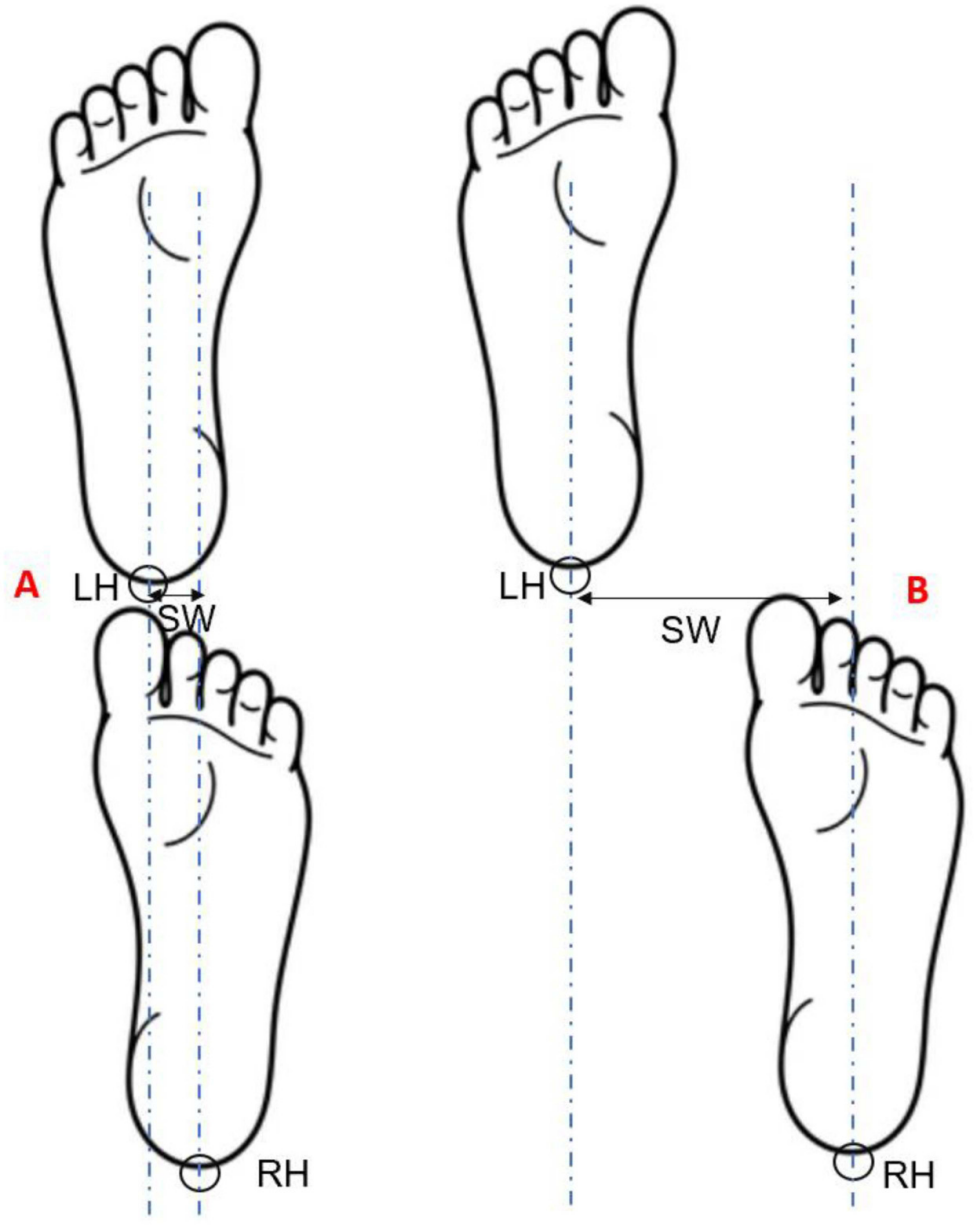
Schematic illustration of step width calculation during tandem walking. **(A)** shows a trial with a relatively narrow step width, while **(B)** shows a trial with a wider step width. LH, left heel marker; RH, right heel marker; vertical dashed lines represent the positions of the heel markers at heel strike; SW, step width, indicated by the double-headed arrow.

Step width, defined as the mediolateral distance between the heels at heel strike (Figure 1), was calculated for each consecutive step throughout the 15-second tandem walking trial. While there is no universally accepted cutoff for a “failed” step during tandem walking, prior work in cerebellar ataxia suggests that step widths near 50 mm reflect instability and deviation from ideal tandem foot placement of 4 mm in healthy controls (28). In this case report, step width was treated as a continuous indicator of balance control, with larger values indicating greater mediolateral instability. All motion trajectory data were processed using a custom-written MATLAB program (MathWorks Inc., Natick, MA, USA) and low-pass filtered at 20 Hz using a second-order Butterworth filter.

The step widths for each heel strike at baseline and at the 1-year follow-up were calculated and summarized descriptively. Because the participant completed only four full steps at baseline and two full steps at follow-up, the sample size was too small to assume normality or perform inferential testing with confidence. Therefore, statistical analyses were de-emphasized, and the focus was placed on descriptive comparisons of the trajectories, mean values, and variability of step width between the two sessions. Observed features of the trajectories (e.g., ability to achieve true tandem stance, corrective steps, and side-to-side differences) were also qualitatively interpreted to provide a more comprehensive picture of the participant's performance.

## Results

**Figure 2:**
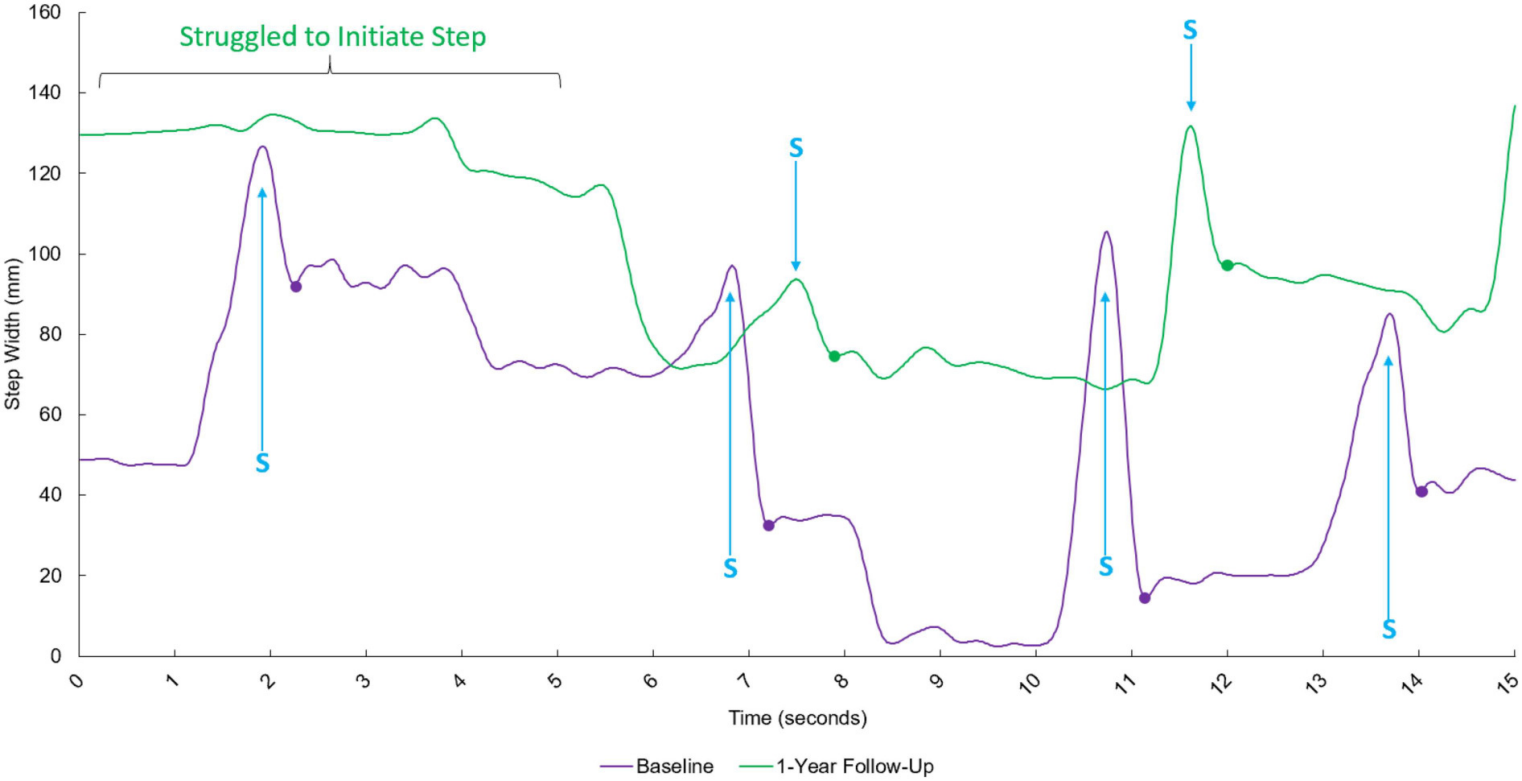
Time-series data exhibiting the step width between the left and right heel during tandem walking for the baseline trial (purple line) and the 1-year follow-up (green line). The *x*-axis represents time after starting in seconds, and the *y*-axis represents the step width between the left and right heel. S = Peak step width during swing phase of stepping foot. Each dot represents the measured step width at the initial foot position and subsequent heel strikes during tandem gait.

The individual took four full steps in the baseline tandem 15-second walking trial and two full steps in the 1-year follow-up. The mean step width during the baseline tandem walking trial was 45.22 ± 33.47 mm (SD). The mean step width at the 1-year follow-up trial was 85.79 ± 15.80 mm (SD) (Figure 2). At the one-year follow-up, the patient was indeed incapable of achieving a true tandem position even in standing, whereas one year earlier they were able to attain a true tandem position, albeit with a corrective step. In addition to the widening of step width, the number of steps achieved within the fixed 15-second trial window decreased from four steps at baseline to two steps at follow-up.

## Discussion

This pilot case report investigated whether tandem walking could detect progressive gait deficits at a one-year period in an individual with FXTAS. In support with our hypothesis, we found an increase in step width during the tandem walking trial at the one-year follow-up compared to baseline.

The observed increase in step width is consistent with known gait characteristics of cerebellar ataxias and supports the notion that individuals with FXTAS exhibit progressive deterioration in mediolateral balance control over time. Tandem walking is a task that challenges dynamic postural stability by narrowing the base of support and requiring precise coordination of foot placement. The increase in step width observed in this case report may represent a compensatory strategy to maintain balance in the face of declining cerebellar control, which is a finding that aligns with prior studies in cerebellar ataxias reporting widened step width and increased gait variability as markers of progression (32, 33). Related cerebellar ataxias, such as spinocerebellar ataxia, have identified changes in tandem gait over a one year period, suggesting that FXTAS may follow a similar trajectory (34). The feasibility of tracking longitudinal changes over time have been observed in individuals with Parkinson's Disease. It is reported that individuals with Parkinson's Disease exhibited a 6%–10% increase in step width for each year that individuals were diagnosed with Parkinson's Disease (35). Lastly, it is reported that aging is significantly correlated to increases in step width during tandem gait, however healthy older adults did not exhibit greater than 50 mm (36). At baseline, our participant may have exhibited a step width that is considered healthy but exceeded the expected step width due to progression of disease.

Tandem walking is a particularly valuable task for assessing cerebellar dysfunction because it requires precise mediolateral control and challenges postural stability by narrowing the base of support. Importantly, tandem walking is already embedded in validated clinical tools such as the Scale for the Assessment and Rating of Ataxia (SARA), highlighting its established acceptability to both patients and clinicians. However, the categorical scoring used in SARA (based on the number of consecutive tandem steps achieved) may lack sensitivity to subtle longitudinal changes in performance. By quantifying kinematic features of tandem walking, such as step width, our case report demonstrates how biomechanical metrics can enhance the resolution of this established clinical task. This approach not only aligns with current clinical practice but also provides a path forward for developing more sensitive markers of progression in FXTAS.

The decline in the number of achievable tandem steps is a complementary indicator of progression. At baseline, the participant could generate four consecutive tandem steps, suggesting that despite instability he could still initiate and maintain the task. At the one-year follow-up, only two steps were possible, reflecting a diminished ability to coordinate successive foot placements and sustain the trial. Taken together with the increased step width, this reduction in step count highlights the progressive nature of mediolateral gait instability in FXTAS.

Several limitations should be considered when interpreting the findings of this pilot case report. First, the case report involved only a single individual with FXTAS, limiting the generalizability of the results. While the within-subject longitudinal design allows for observation of change over time, individual variability in disease trajectory, compensatory strategies and physical condition may not reflect patterns observed in the broader FXTAS population. Second, only one 15-second tandem walking trial was conducted at each time point, which may reduce the reliability of the measured outcomes and increase the chance of random variability. Repeated trials, and increased steps such as SARA clinical guidelines, would improve measurement stability and increase the sensitivity to detect subtle changes. Additional kinematic variables such as step length variability, trunk sway, or center of mass excursions may provide a more comprehensive understanding of motor decline. Finally, potential confounding factors such as fatigue, attention (37–40), medication status (41), and motivation at the time of testing were not controlled, which could influence gait performance in a single-session assessment. Lastly, the disease stage of FXTAS in our participant was not clinically classified, and no biomechanical staging system currently exists for this condition, although the FXTAS clinical stage is determined by the severity of the motor problems and their interference with activities of daily living (42). Consequently, the baseline and follow-up assessments may have coincided with an atypical or non-representative phase of disease progression. Future studies should aim to recruit individuals with genetically confirmed FXTAS in early disease stages or *FMR1* premutation carriers who have not developed FXTAS who exhibit relatively preserved gait function at baseline and track them longitudinally to enable more meaningful interpretations of functional decline and clinically relevant disease progression.

Comparative case studies in rare progressive gait disorders further highlight the utility of targeted kinematic and biomechanical analysis. For instance, Sassi et al. described altered ankle muscle activity and gait propulsion deficits in siblings with progressive pseudorheumatoid dysplasia, using EMG and kinematics to elucidate compensatory patterns during stance (43). In another case, Tedeschi et al. combined spatial-temporal parameters with plantar pressure mapping in a patient with Strumpell–Lorrain disease, identifying monopodal reliance and increased forefoot loading due to dorsiflexion constraints (44). Although our case report employs a simpler tandem walking paradigm, focusing specifically on mediolateral stability through step width, these reports highlight the depth and range of gait features that can be revealed with more comprehensive approaches. Such multi-modal assessments represent promising directions for future longitudinal gait research in FXTAS and related ataxias.

In conclusion, this feasibility case report demonstrates that step width during tandem walking increased markedly over one year in an individual with FXTAS, suggesting that this measure may be a biomarker for motor progression. Future studies should incorporate additional gait variables such as cadence and stride time variability, foot placement asymmetry, or stance to swing ratios. Most importantly, this case report provides vital feasibility data to examine the progression of FXTAS across multiple disease stages.

## Data Availability

The raw data supporting the conclusions of this article will be made available by the authors, without undue reservation.
